# Predictors and prognosis of early ischemic mitral regurgitation in the era of primary percutaneous coronary revascularisation

**DOI:** 10.1186/1476-7120-12-14

**Published:** 2014-04-03

**Authors:** Jimmy MacHaalany, Olivier F Bertrand, Kim O’Connor, Eltigani Abdelaal, Pierre Voisine, Éric Larose, Éric Charbonneau, Olivier Costerousse, Jean-Pierre Déry, Mario Sénéchal

**Affiliations:** 1 Department of Cardiology, Institut Universitaire de Cardiologie et de Pneumologie de Québec, 2725 chemin Sainte-Foy, G1V 4G5 Quebec City, Quebec, Canada; 2 Department of Cardiovascular Surgery, Institut Universitaire de Cardiologie et de Pneumologie de Québec, Quebec City, Quebec, Canada

**Keywords:** Ischemic mitral regurgitation, Mitral valve, Primary percutaneous coronary intervention, Myocardial infarction

## Abstract

**Background:**

Studies assessing ischemic mitral regurgitation (IMR) comprised of heterogeneous population and evaluated IMR in the subacute setting. The incidence of early IMR in the setting of primary PCI, its progression and clinical impact over time is still undetermined. We sought to determine the predictors and prognosis of early IMR after primary percutaneous coronary intervention (PCI) for ST-elevation myocardial infarction (STEMI).

**Methods:**

Using our primary PCI database, we screened for patients who underwent ≥2 transthoracic echocardiograms early (1–3 days) and late (1 year) following primary PCI. The primary outcomes were: (1) major adverse events (MACE) including death, ischemic events, repeat hospitalization, re-vascularization and mitral repair or replacement (2) changes in quantitative echocardiographic assessments.

**Results:**

From January 2006 to July 2012, we included 174 patients. Post-primary PCI IMR was absent in 95 patients (55%), mild in 60 (34%), and moderate to severe in 19 (11%). Early after primary PCI, IMR was independently predicted by an ischemic time > 540 min (OR: 2.92 [95% CI, 1.28 – 7.05]; p = 0.01), and female gender (OR: 3.06 [95% CI, 1.42 – 6.89]; p = 0.004). At a median follow-up of 366 days [34–582 days], IMR was documented in 44% of the entire cohort, with moderate to severe IMR accounting for 15%. During follow-up, MR regression (change ≥ 1 grade) was seen in 18% of patients. Moderate to severe IMR remained an independent predictor of MACE (HR: 2.58 [95% CI, 1.08 – 5.53]; p = 0.04).

**Conclusions:**

After primary PCI, IMR is a frequent finding. Regression of early IMR during long-term follow-up is uncommon. Since moderate to severe IMR post-primary PCI appears to be correlated with worse outcomes, close follow-up is required.

## Introduction

Functional ischemic mitral regurgitation (IMR) has been described after acute myocardial infarction (MI). However, its prevalence and spectrum of severity has constantly varied in previously published studies (1–18). This disparity is explained by the different methods utilized for assessing IMR, the heterogeneity of studied populations, and different reperfusion utilized techniques [1-18]. In addition, some of this data is based on secondary analysis of clinical trials [1,9,10,12,13], which are subject to referral and selection biases, while others assessed and quantified the severity of IMR in the subacute time period [2-6,16,17]. Finally, of these studies, only a minority was based on modern primary PCI strategy [3,9,15,18].

The presence of IMR is clinically relevant as it has been associated with poorer clinical outcomes [6,10,13] and can lead to a worse prognosis in patients undergoing coronary artery bypass grafting (CABG) [19]. Primary PCI has become the preferred method for reperfusion therapy in STEMI as it significantly reduces negative left ventricular remodeling, lowers complication rates and improves survival [20-22]. Some recent data has also suggested that primary PCI could decrease the incidence of IMR after STEMI [3].

Our study primary aim was to address the aforementioned gaps by determining the predictors of early IMR, and to evaluate the prognostic value of IMR after primary PCI. Additionally, we sought to evaluate the prevalence and severity of early IMR in the acute phase of STEMI treated with primary PCI and to assess the echocardiographic changes in quantitative IMR over one year.

## Methods

### Patient population and follow-up

From January 2006 to July 2012, 174 consecutive patients who were referred to our tertiary care university center for primary PCI within 12 h after symptom onset, who underwent early (1–3 days) and late echocardiographic studies, and had appropriate clinical follow-up at our center we retrospectively included. Exclusion criteria were: 1) previous CABG, 2) STEMI patients without any significant coronary lesion or in whom recent fibrinolytic therapy was administered, 3) patients with any valvular pathology other than MR or mitral valve papillary muscle rupture and 4) patients with incomplete echocardiographic data. Cardiac catheterization was performed via radial approach using 5–6 Fr-guiding catheters, with every patient receiving aspirin and clopidogrel. Adjunctive pharmacotherapy, such as bivalirudin or glycoprotein IIb/IIIa inhibitors was left to the operator’s discretion and was similar between all groups. All patients gave informed written consent. This study was performed in accordance with our institution review board and ethics committee. This study is conform to ethical committee rules of Institut universitaire de cardiologie et de pneumologie de Québec, Québec, Canada.

IMR was classified from analysis of clinical information, operative reports, and echocardiograms. MR was judged to be ischemic in origin when the valve leaflets and chordae were normal and the regurgiation was caused as a consequence of the STEMI. For analysis, patients were stratified according to their baseline IMR after primary PCI. Baseline data was obtained from a computerized medical database of prospectively recorded demographic, clinical and procedural information. Clinical follow-up information was obtained from the referring physicians or via direct phone contact with patients. Finally, information on vital status at 1 year was also collected from the Quebec death registry “Directeur de l’État Civil” Quebec service counter.

### Echocardiographic analysis

Two-dimensional and Doppler transthoracic echocardiography examinations were performed using Philips Medical Systems (Amsterdam, Netherlands) platforms. The two-dimensional echocardiograms were analyzed by two experienced investigators (J.M. and M.S.) via the Xcelera Echo Lab Management (Amsterdam, Netherlands). The inter-observer reliability, based on the interclass correlation coefficient, for all echocardiographic data were very good with absolute values higher than 0.87.

All patients had early post-PCI (1–3 days) (Echo 1), and long-term follow-up (median: 244 days [85 – 533 days] (Echo 2)) echocardiographic studies. Left ventricular ejection fractions (LVEF) and volumes were averaged values from the apical 4-chamber and 2-chamber views and calculated using the modified biplane Simpson method. LV dimensions were measured using M-mode technique via the parasternal-long axis view. LV sphericity index was calculated by dividing the LV short-axis dimension by the LV long-axis dimension in the 4-chamber view. Left atrial (LA) size was assessed by averaging the LA volume measured in 4-chamber and 2-chamber views with the use of 2-dimensional planimetry [23]. Pulmonary artery pressure (PASP) was calculated as per recommendations. Mitral regurgitation was quantified initially using color flow Doppler, adding supportive signs and quantitative parameters (predominantly vena contracta (VC)) and according to the American Society of Echocardiography guidelines [24]. In cases of discrepancy between the methods as to the grade of MR, the available quantitative parameter was used as the reference technique.

### Outcomes

*Procedural success* was defined as a final Thrombolysis In Myocardial Infarction flow of 3 with a residual stenosis of <20%. *Death* was defined as all-cause mortality. *Re-hospitalization* was defined as hospital admissions for acute heart failure. *Myocardial infarction* definition was based on the previously published universal definition of myocardial infarction [25]. The rate of *major adverse cardiac events (MACE)* was the composite of death, MI, stroke, re-hospitalization for congestive heart failure, PCI or CABG and mitral repair or replacement.

### Statistical analysis

Categorical variables were expressed as numbers and percentages and continuous variables as mean ± SD or medians with interquartile range [IQR]. Differences between groups were assessed using ANOVA for continuous variables, and the Pearson’s χ^2^ test test for categorical variables as appropriate. The Cox proportional hazard model was used to identify independent predictors. Potential predictors of post-PCI IMR severity and MACE were chosen from patient’s baseline clinical, procedural and echocardiographic characteristics. IMR grade was chosen as a categorical value. Variables were selected with stepwise, backward, and forward procedures with logistic regression analyses, which were entered into the model at p < 0.10 and retained at p < 0.05. The cutoff values relating to baseline LVESD, and ischemic time prior to revascularization procedure were based on ROC curve analysis. Survival curves were constructed using Kaplan-Meier methods with comparisons made using the log-rank test. A probability value of < 0.05 was considered significant and all calculations and statistical tests were performed using JMP statistical software version 10.0.0 (SAS institute, Cary, NC).

## Results

Clinical follow-up was complete in all patients. Overall mean age was 63 ± 12 years. Majority of the 174 patients (79%) were males and clinical median follow-up was 366 days [IQR: 34 – 582 days]. Patients with moderate to severe IMR involved more patients aged > 65 years (p = 0.046) and more women (p = 0.006) (Table 1). There was also a progressive increase in ischemic time prior to PCI according to MR severity (297 ± 23 min for no MR vs. 301 ± 28 min for mild MR vs. 486 ± 52 min for moderate or severe MR; p = 0.004) (Table 2). Angiographic parameters prior and after primary PCI were similar between groups.

**Table 1:** Baseline characteristics

**Variable**	**ALL (n = 174; 100%)**	**No MR (n = 95; 55%)**	**Mild MR (n = 60; 34%)**	**Moderate or severe MR (n = 19; 11%)**	**P value**
Age (yrs)	63 ± 12	61 ± 1	63 ± 2	66 ± 3	0.21
Age > 65	73 (42%)	37 (39%)	23 (38%)	13 (68%)	0.046
Male sex	138 (79%)	83 (87%)	44 (73%)	11 (58%)	0.006
Weight (kg)	82 ± 56	86 ± 6	78 ± 7	78 ± 13	0.43
Height (cm)	170 ± 8	171 ± 1	170 ± 1	166 ± 2	0.08
Body mass index (kg/m^2^)	28 ± 21	30 ± 2	27 ± 3	28 ± 5	0.33
Body surface area (m^2^)	1.94 ± 0.37	1.96 ± 0.03	1.91 ± 0.05	1.90 ± 0.09	0.57
Diabetes mellitus	19 (11%)	9 (10%)	7 (12%)	3 (16%)	0.70
Hypertension	83 (48%)	41 (43%)	31 (52%)	11 (58%)	0.38
Current smoking	64 (37%)	41 (43%)	21 (35%)	2 (11%)	0.03
Dyslipidemia	81 (47%)	38 (40%)	32 (53%)	11 (58%)	0.15
Stable angina	12 (7%)	4 (4%)	5 (8%)	3 (16%)	0.17
Unstable angina	4 (2%)	2 (2%)	1 (2%)	1 (5%)	0.65
Prior MI	17 (10%)	9 (10%)	5 (8%)	3 (16%)	0.63
Prior PCI	13 (9%)	4 (4%)	6 (10%)	3 (16%)	0.18
Creatinine clearance (mL/min)†	84 ± 49	89 ± 5	79 ± 7	72 ± 11	0.30
GFR > 60 mL/min	128 (78%)	73 (83%)	40 (69%)	15 (79%)	0.14
Follow-up (days)	366 [34–582]	476 ± 42	455 ± 52	364 ± 93	0.49

†Based on the the Cockroft-Gault equation.

MR: Mitral Regurgitation; MI - Myocardial infarction; PCI - Percutaneous intervention; eGFR - Estimated glomerular filtration rate.

**Table 2:** Angiographic characteristics

**Variable**	**ALL (n = 174; 100%)**	**No MR (n = 95; 55%)**	**Mild MR (n = 60; 34%)**	**Moderate or severe MR (n = 19; 11%)**	**P value**
Ischemic time (min)*	319 ± 226	297 ± 23	301 ± 28	486 ± 52	0.004
Thrombectomy catheter	104 (60%)	59 (62%)	35 (59%)	10 (53%)	0.73
Multi-vessel disease^¶^	88 (52%)	43 (45%)	34 (57%)	13 (68%)	0.12
Culprit vessel					
Left anterior descending	83 (48%)	49 (52%)	28 (47%)	6 (32%)	0.28
Left circumflex	27 (16%)	14 (15%)	9 (15%)	4 (21%)	0.78
Right coronary artery	63 (36%)	32 (34%)	22 (37%)	9 (47%)	0.52
TIMI Flow – pre-stenting					
0	73 (42%)	38 (40%)	26 (43%)	9 (47%)	0.81
1	73 (42%)	39 (41%)	27 (45%)	7 (37%)	0.79
2	11 (6%)	8 (8%)	2 (3%)	1 (5%)	0.44
3	17 (10%)	9 (10%)	6 (10%)	2 (11%)	0.99
0 or 1	146 (84%)	77 (81%)	53 (88%)	16 (84%)	0.49
TIMI Flow – post- stenting					
0	1 (1%)	0 (0%)	1 (2%)	0 (0%)	0.39
1	3 (2%)	0 (0%)	3 (5%)	0 (0%)	0.06
2	26 (15%)	16 (17%)	7 (12%)	3 (16%)	0.68
3	143 (83%)	79 (83%)	49 (82%)	16 (84%)	0.96
0 or 1	4 (2%)	0 (0%)	4 (7%)	0 (0%)	0.02
Angiographic success^†^	149 (86%)	82 (86%)	50 (83%)	17 (90%)	0.77
Peak CK-MB (μg/L) (ug/L)	173.4 [56.0 – 378.8]	151 [56–365]	212 [77 – 373]	163 [6.9 – 481]	0.66
Peak troponin T (μg/L)	4.7 [2.2 – 8.9]	4.5 [2.4 – 7.9]	5.1 [2.2 – 9.1]	5.9 [1.7 – 11.4]	0.45

*Ischemic time: defined as the time difference between beginning of the chest pain and artery opening.

^¶^Multivessel disease: defined as significant coronary artery disease (>50% diameter stenosis) in ≥ 2 major epicardial artery.

^†^Angiographic success: defined as <20% residual stenosis + TIMI 3 flow post-PCI.

MR: Mitral Regurgitation; TIMI - Thrombolysis in myocardial infarction; CK-MB - Serum myocardial band of creatine kinase.

### Echocardiographic characteristics

Early after primary PCI, IMR was absent in 95 patients (55%), mild in 60 (34%), moderate or severe in 19 (11%). Left-ventricular systolic dimension (LVESD) increased in a graded relationship with aggravation of IMR (3.4 cm ± 0.1 vs. 3.6 cm ± 0.1 vs. 3.8 cm ± 0.1; p = 0.02), translating into lower LVEF (48% ± 1 vs. 45% ± 2 vs. 41% ± 3; p = 0.03) (Table 3).

**Table 3:** Echocardiographic characteristics at baseline and follow-up

**Variable**	**ALL (n = 174; 100%)**	**No MR (n = 95; 55%)**	**Mild MR (n = 60; 34%)**	**Moderate or severe MR (n = 19; 11%)**	**P value**
***Baseline (POST-PCI)***					
LVEDD (mm)	47 ± 6	46 ± 1	47 ± 1	48 ± 1	0.40
LVESD (mm)	35 ± 7	34 ± 1	36 ± 1	38 ± 2	0.02
LVESD >40 mm	39 (22%)	15 (16%)	18 (30%)	6 (32%)	0.07
LVEDV (ml)	102 ± 35	100 ± 4	104 ± 5	107 ± 8	0.68
LVESV (ml)	57 ± 29	53 ± 3	59 ± 4	66 ± 7	0.16
LVEF (%)	46 ± 12	48 ± 1*	45 ± 2*	41 ± 3	0.03
LV sphericity – diastole (%)	0.55 ± 0.07	0.54 ± 0.01	0.56 ± 0.01	0.56 ± 0.02*	0.29
LA volume - indexed (ml/m^2^)	26 ± 9	23 ± 1*	28 ± 1	36 ± 2	<0.0001
MR – Vena Contracta (cm)	0 [0 – 0.23]	0 [0 – 0]*	0.19 [0.10 – 0.27]	0.62 [0.53 – 0.7]*	<0.0001
PASP (mmHg)	29 ± 10	26 ± 1	29 ± 1	43 ± 3	<0.0001
***Long-term follow-up***					
LVEDD (mm)	48 ± 9	48 ± 1	48 ± 1	51 ± 2	0.34
LVESD (mm)	35 ± 10	34 ± 1	35 ± 1	39 ± 2	0.11
LVEDV (ml)	114 ± 47	111 ± 5	116 ± 6	120 ± 10	0.71
LVESV (ml)	59 ± 40	56 ± 4	60 ± 5	72 ± 9	0.27
LVEF (%)	52 ± 13	53 ± 1*	52 ± 2*	44 ± 3	0.02
LV sphericity – diastole (%)	0.57 ± 0.09	0.55 ± 0.01	0.57 ± 0.01	0.61 ± 0.02*	0.01
LA size - indexed (ml/m^2^)	30 ± 12	27 ± 1*	30 ± 2	41 ± 3	<0.0001
MR – Vena Contracta (cm)	0.1 [0 – 0.23]	0 [0 – 0.19]*	0.12 [0 – 0.24]	0.54 [0.44 – 0.6]*	<0.0001
PASP (mmHg)	28 ± 10	26 ± 1	27 ± 1	36 ± 2	0.001

*Difference between baseline and long-term follow-up values is statistically significant; (all p-values ≤ 0.03).

LVEDD – Left ventricular end-diastolic diameter; LVESD – Left ventricular end-systolic diameter; LVEDV – Left ventricular end-diastolic volume; LVESV – Left ventricular end-systolic volume; LVEF – Left Ventricular Ejection Fraction; LA – Left atrium; MR- Mitral regurgitation; PASP – Pulmonary artery systolic pressure.

**Figure 1:**
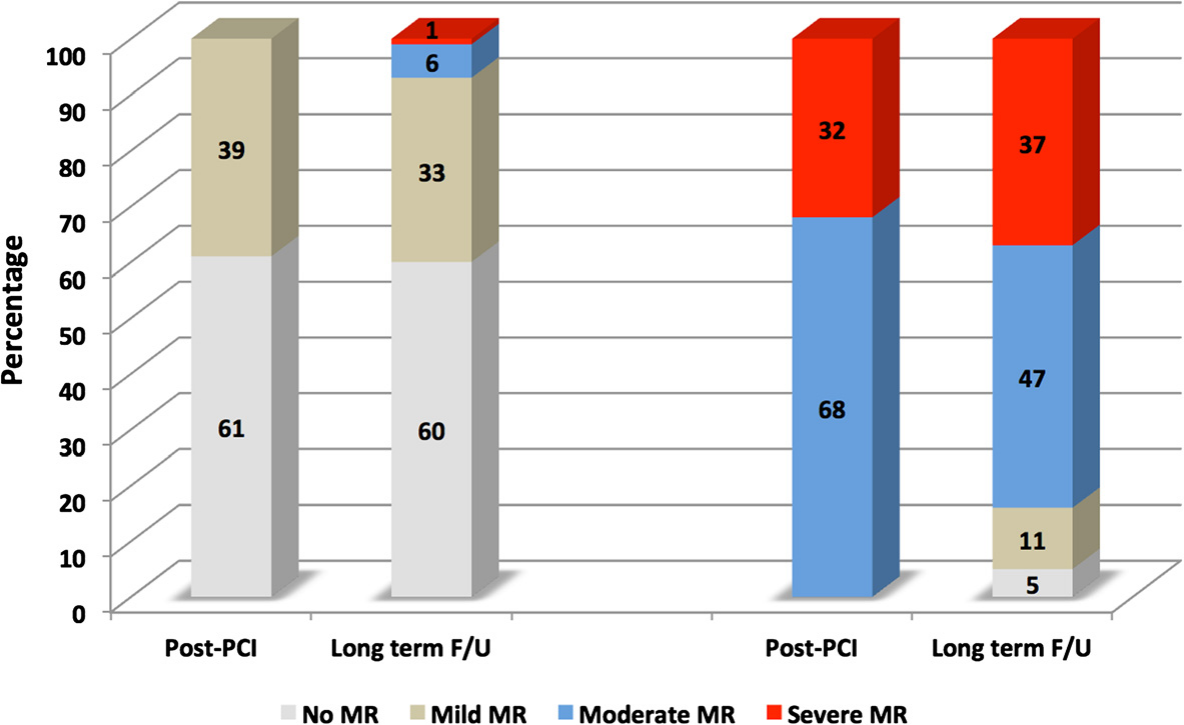
Distribution of ischemic functional mitral regurgitation (MR) over time according to baseline MR grade.

The echocardiographic studies median follow-up was 244 days [85 – 533 days], with the moderate to severe IMR incidence accounting for 15%. No differences between groups were observed at the end of follow-up with respect to diastolic and systolic echocardiographic parameters. A lower LVEF was observed as the severity of IMR increased (53% ± 1 for no MR vs. 52% ± 2 for mild MR vs. 44% ± 3 for moderate or severe MR; p = 0.02), with the left ventricular sphericity increasing significantly over time only in the moderate to severe MR group (Table 3). IMR progressed or regressed (by ≥ 1 grade) in 38 (22%) and 32 (18%) patients, respectively. Analysis of patient’s according to their baseline MR grade is presented in Figure 1. Seven percent of patients (n = 11) with clinically no or mild IMR progressed to a moderate or severe grade, while 16% (n = 3) regressed from moderate or severe to no or mild grade.

### Clinical outcomes

**Figure 2:**
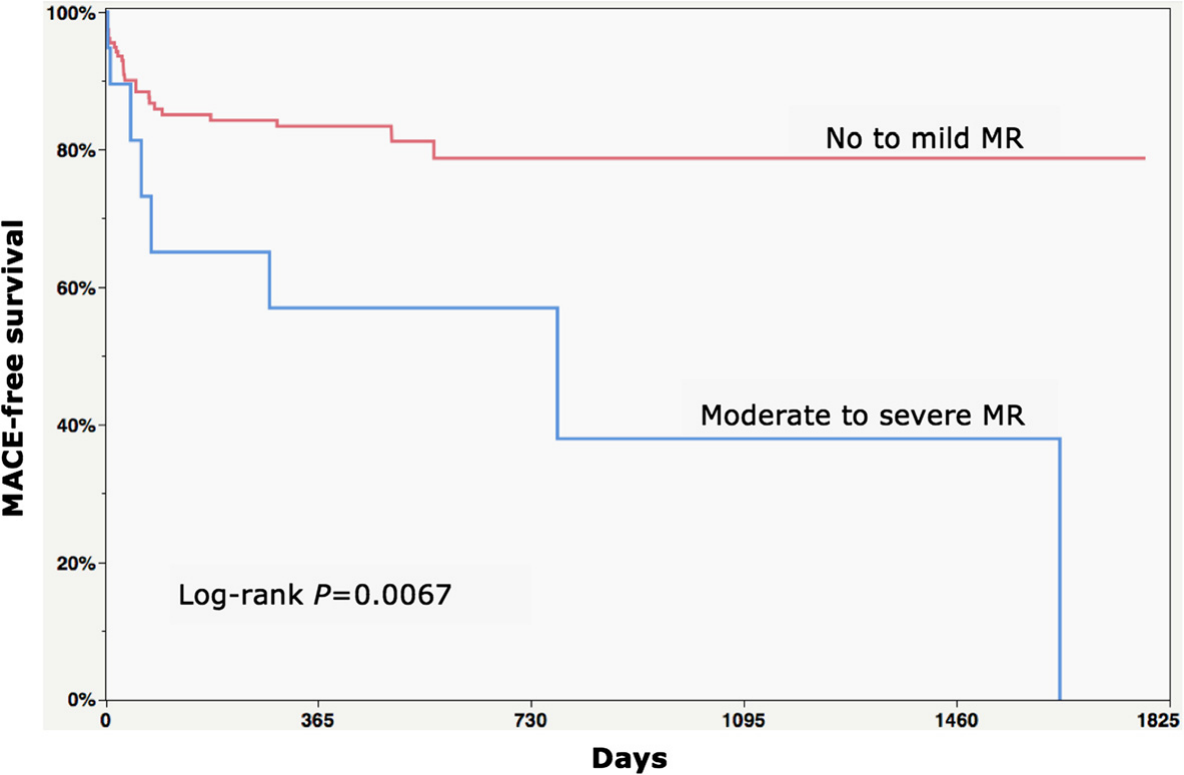
Kaplan-Meier curves for the combined endpoint of MACE (death, MI, stroke, re-hospitalization for congestive heart failure, PCI or CABG and mitral repair or replacement).

No difference was observed in early (≤ 30 days) mortality (0% vs. 2% vs. 0%; p = 0.39) and CABG revascularization (2% vs. 0% vs. 5%; p = 0.28) between all 3 groups. The composite MACE endpoint was observed more frequently within the moderate or severe group (16% vs. 17% vs. 42%; p = 0.02) (Table 4 and Figure 2). This was mainly driven by a higher number of surgical revascularization procedures (CABG - no-MR: 6% vs. mild MR: 7% vs. moderate or severe MR: 26%; p = 0.008) with concomitant mitral valve intervention (no-MR: 0% vs. mild MR: 0% vs. moderate or severe MR: 26%; p < 0.0001).

**Table 4:** Cumulative clinical outcomes according to early ischemic MR severity

**Variable**	**All (n = 174; 100%)**	**No MR (n = 95; 55%)**	**Mild MR (n = 60; 34%)**	**Moderate or severe MR (n = 19; 11%)**	**P value**
Death	2 (1**%**)	0 (0**%**)	1 (2**%**)	1 (5**%**)	0.13
Stroke	3 (2**%**)	2 (2**%**)	1 (2**%**)	0 (0**%**)	0.81
Re-infarction	13 (8**%**)	10 (11**%**)	2 (3**%**)	1 (5**%**)	0.23
Re-Hospitalization for CHF	2 (1**%**)	0 (0**%**)	1 (2**%**)	1 (5**%**)	0.13
Percutaneous coronary intervention	9 (5**%**)	6 (6**%**)	2 (3**%**)	1 (5**%**)	0.72
Coronary artery bypass grafting	14 (8**%**)	5 (6**%**)	4 (7**%**)	5 (26**%**)	0.008
Annuloplasty or mitral valve surgery	5 (3**%**)	0 (0**%**)	0 (0**%**)	5 (26**%**)	<0.0001
MACE*	33 (19**%**)	15 (16**%**)	10 (17**%**)	8 (42**%**)	0.02

*Major adverse cardiac events (MACE): defined as a combined endpoint of death, MI, stroke, re-hospitalization for congestive heart failure, PCI or CABG and mitral repair or replacement.

MR: Mitral Regurgitation; CHF: Congestive Heart Failure; MACE: Major Adverse Cardiovascular Event.

**Figure 3:**
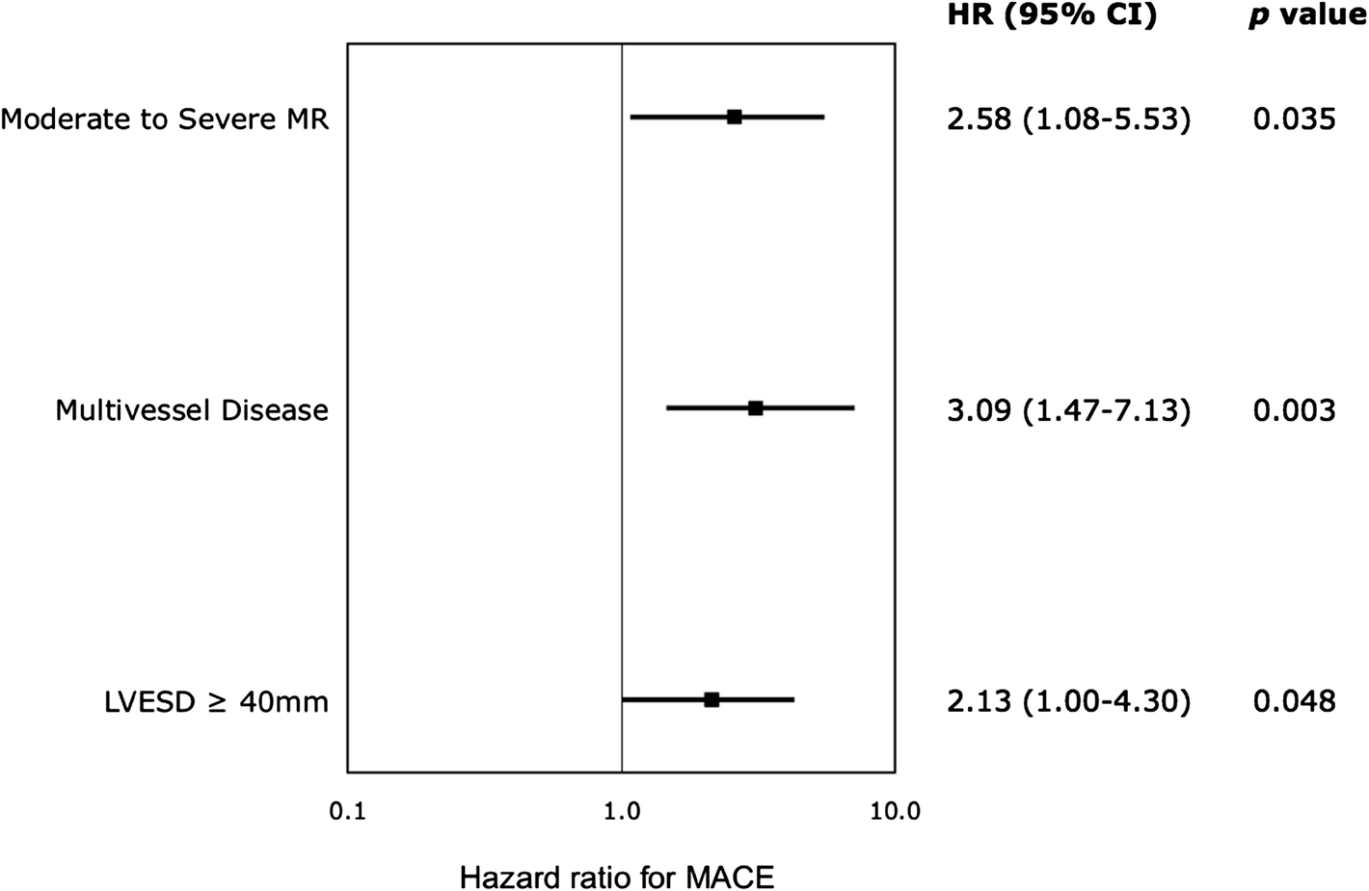
Independent predictors for the combined endpoint of MACE (death, MI, stroke, re-hospitalization for congestive heart failure, PCI or CABG and mitral repair or replacement).

From the multivariate analysis, we found that IMR early after primary PCI was independently predicted by an ischemic time prior to PCI > 540 min (OR: 2.92 [95% CI, 1.28 – 7.05]; p = 0.01), and female gender (OR: 3.06 [95% CI, 1.42 – 6.89]; p = 0.004). Furthermore, moderate to severe MR was a strong independent predictor of 1-year MACE (HR: 2.58 [95% CI, 1.08 – 5.53]; p = 0.04). Other independent predictors of 1-year MACE included multivessel disease (HR: 3.09 [95% CI, 1.47 – 7.13]; p = 0.003) and LVESD at baseline ≥ 40 mm (HR: 2.13 [95% CI, 1.00 – 4.30]; p = 0.05) (Figure 3).

## Discussion

After primary PCI for acute STEMI within 12 hours of symptom onset, we found that: 1) moderate to severe IMR by quantitative echocardiographic analysis is apparent in 11% of patients acutely and remains evident in 15% after a median follow-up of 1 year; 2) female gender, and longer ischemic time are strong independent predictors of early significant IMR; 3) IMR could worsen in patients with evidence of none or mild IMR at baseline and usually will not regress in those with significant MR; 4) the presence of moderate or severe IMR, multivessel disease or an LVESD at baseline ≥ 40 mm predict patients worse late clinical outcomes; 4) Importantly, patients with moderate to severe IMR experience a higher incidence of one year MACE, which is mainly driven by higher rates of surgical mitral valve revascularization with concurrent valvular correction.

Functional IMR has an important prognostic impact after myocardial infarction. It is generated by papillary muscles displacement secondary to the infarcted LV [26]. Due to the non-extensibility of the chordae, papillary muscle translation exerts traction on leaflets through strut chordae implanted on the leaflets body [27,28]. This produces tethering with apical and lateral leaflets displacement [26,29], annular flattening and enlargement, all elements that converge to yield functional IMR. The prevalence has been variable (Table 5) as it is dependent on the detection technique utilized. It ranges from <20% in angiographic studies [10-14] to as high as 60% in echocardiographic studies [1-8,15,30-33], with moderate or severe IMR accounting for <5% and 10-15% of cases, respectively. This discrepancy reflects the selection biases inherent to most studies, as most involved a heterogeneous sample size that included a mixture of STEMI and NSTEMI patients. Some were exposed to selection bias as they were based on sub-analysis of larger randomized trials [1,10,12,13] while others were samples of patients admitted to the coronary care unit. Furthermore, various studies were solely based on thrombolytic therapy [1,10,12-14,32,33], which is recognized as a less effective treatment in STEMIs vs. primary PCI [34], and ultimately not everyone excluded patients with previous MI [2-4,32]. Our study attempted to take into consideration most of these issues by selecting a homogenous sample population composed of all-comers presenting to the catheterization laboratory in the setting of primary PCI setting, and in the first 12 hours of symptoms onset. Additionally, these patients were all treated within today’s standard of care.

**Table 5:** Comparison between echocardiographic studies examining the prevalence of ischemic mitral regurgitation in STEMI population

**Author (Enrollment years)**	**N**	**MR prevalence (%)**	**Moderate or severe MR (%)**	**Treatment strategy**	**STEMI**	**F/U**	**Death (%)**	**Other**	**Mitral surgical correction (%)**
Barzilai et al. [31]**(1985-1986)**	59	39	NR	NR	59**%**	14 m	MR (+): 48**%**, MR (0): 11**%**	NR	NR
Bursi et al. [2]**(1988-1998)**	773	50	12	NR	59**%**	5 y	None: 28**%**, Mild: 38**%**, M-S: 60**%**	**CHF Adm**	NR
								None: 16**%**, Mild: 26**%**, M-S: 65**%**	
Grigioni et al. [6]**(1990-1997)**	303	64	NR	NR	100**%**	5 y	MR (+): 62**%**, MR (0): 39**%**	**Card Death**	NR
								MR (+): 50**%**, MR (0): 30**%**	
Amigoni et al. (Valiant study) [1]**(1998-2001)**	496	53	13	Thrombolytic (40**%**), PCI (19**%**)	63**%**	20 m	None: 10**%**, Mild: 21**%**, M-S: 30**%**	NR	NR
Feinberg et al. [5]**(1996)**	417	35	6	Thrombolytic (42**%**), PTCA / CABG (41**%**)	100**%**	1 y	None: 4.8**%**, Mild: 12.4**%**, M-S: 24**%**	NR	NR
Van Dantzig et al. [33]**(1991-1993)**	188	NR	13	Thrombolytic (55**%**)	62**%**	NR	NR	NR	NR
Leor et al. [32]**(N/A)**	104	33	11	Thrombolytic (100**%**)	100**%**	1 m	NR	NR	NR
Aronson et al. [30]**(2000-2005)**	1190	46	6	PCI (30**%**)	100**%**	2 y	None: 8**%**, Mild: 15**%**, M-S: 38**%**	**CHF Adm**	NR
								None: 0.6**%**, Mild: 3.6**%**, M-S: 4**%**	
Barra et al. [16]**(2006-2008)**	796	45	15	PCI (56**%**)	45**%**	2 y	None: 12**%**, Mild: 22**%**, M-S: 39**%**	**CHF Adm**	NR
								None: 11**%**, Mild: 24**%**, M-S: 20**%**	
Chung et al. [4]**(2000-2002)**	519	56	15	PCI (81**%**)	86**%**	6 m	None - Mild: 10**%**, M-S: 19**%**	NR	NR
Chua et al. [3]**(2000-2004)**	318	17	NR	PCI (100**%**)	100**%**	7 y	MR (+): 49**%**, MR (0): 26**%**	NR	NR
Wita et al. [15]**(N/A)**	83	42	NR	PCI (100**%**)	100**%**	6 m	NR	NR	NR
Uddin et al. [18]**(2003-2007)**	888	47	11	PCI (100**%**)	100**%**	5 y	None: 13**%**, Mild: 18**%**, M-S: 34**%**	NR	None: 0**%**, Mild: 0**%**, M-S: 4**%**
MacHaalany et al. **(2006–2012)**	174	44	11	PCI (100**%**)	100**%**	1 y	None: 0**%**, Mild: 2**%**, M-S: 5**%**	**CHF Adm**	None: 0**%**, Mild: 0**%**, M-S: 26**%**
								None: 0**%**, Mild: 2**%**, M-S: 5**%**	

MR: Mitral Regurgitation; STEMI: ST elevation Myocardial Infarction; F/U: follow-up; PTCA: Percutaneous transluminal coronary angioplasty; PCI: Percutaneous Coronary Intervention; CABG: Coronary Artery Bypass Grafting; M-S: moderate to severe MR; Card Death: Cardiovascular Death; CHF Adm: Congestive Heart Failure Admission; NR: Not Reported.

In accordance to previous studies [1,7,9,13,30], our data indicate that female gender is an important determinant in the severity of early IMR. This finding may partly provide an explanation for the higher incidence of post-STEMI mortality seen in females [35-37]. We have also found that longer ischemic time prior to PCI is an important determinant in the severity of IMR. This finding once again emphasizes the notion of early opening of the infarcted-artery with the aim of salvaging as much muscle mass as possible; therefore reducing the rate of IMR occurrence.

Previous studies have shown IMR to be an independent predictor of outcome as it doubled mortality rates and increased the incidence of hospital admissions for heart failure [1,6,10,13]. Our data does not suggest large differences in the individual clinical outcomes between all 3 groups. We hypothesize that this difference results specifically from the different timeline in which these studies occurred (early 1980s to late 1990s vs. late 2000s to early 2010s). During this time period, percutaneous approaches and pharmacologic treatments have significantly improved, thus enhancing clinical outcomes. Nonetheless, MACE outcome was higher in the moderate to severe MR group and was mainly driven by surgical coronary revascularization and mitral valve surgical correction >30 days after the index ischemic event. Interestingly, all patients in the moderate to severe MR group who underwent surgical revascularization required mitral surgical correction. This indicates that this group of patients necessitate close follow-up in the first year post-PCI, as in the majority of cases, the moderate to severe MR will not regress, leading to an indication for surgical intervention. Lastly, the presence of moderate to severe MR, multi-vessel coronary artery disease and a large ventricular dimension predicted the development of MACE, all of which are indicative of a higher risk population.

The ACC/AHA and ESC/EACTS guidelines [38,39] provide little and unclear recommendations as for surgical treatment in functional IMR. The only echocardiographic parameter used to guide clinicians is an LVEF ≥ 30% in patients with moderate to severe IMR undergoing CABG that was recently adopted in the ESC/EACTS guidelines [39]. Our data suggest that, similar to degenerative MR, an increase in LVESD (≥ 40 mm) may also be an appropriate clinical marker for poor long-term clinical outcome. Future prospective trials are clearly required to definitely answer this question.

### Limitations

The primary limitation of this study lies in the retrospective nature of data collection. The inherent treatment biases of the physicians caring for the patients could have lead to the introduction of selection bias or unidentified confounding factors that may have influenced our results. Our facility is a tertiary care center that accepts patients from different centers for the primary PCI procedure, and returned to the original center as soon as hemostasis is achieved. This approach might have underestimated the true value of IMR. Our echocardiographic data is observed after the MI and do not exclude that some MR was present beforehand in some patients, a limitation shared by most studies on this topic. Nonetheless, our prevalence exceeds what has been documented in the general population [40] therefore; it is most likely attributed to the MI. A prospective multicenter study evaluating LV remodeling and powered for clinical outcomes should be performed to validate our results.

## Conclusions

In the era of primary PCI for STEMI, early and 1-year post-PCI moderate to severe IMR was documented in 11% and 15% respectively. Female gender, and ischemic time prior to PCI > 540 min strongly predicted the occurrence of IMR while multivessel disease, moderate or severe MR, and LVESD at baseline ≥ 40 mm predicted combined MACE outcome. Moreover, around 25% of patients with moderate to severe IMR required subsequent surgical revascularization with mitral valve surgical correction. Close clinical follow-up is highly recommended in this particular population as indication for surgical intervention may likely arise in the first year post-ischemic event.

## Competing interests

The authors declare that they have no competing interests.

## Authors’ contributions

MS is the principal investigator, he has designed the study, treated the patients, drafted the manuscript, performed the echocardiography's and interpreted the data; JM interpreted the data, did the statistical analysis and participated in the drafting of the manuscript; OFB, EL, KO, PV, EC, JPD and EA treated the patients and has revised the manuscript, OC did the statistical analysis and has revised the manuscript. All the authors read and approved the final manuscript.
